# Supplementary material for: Measuring quality of life in trials including patients on dialysis: how are transplants and mortality incorporated into the analysis? A systematic review protocol
Source: BMJ Open. 2021 Aug 18;11(8):e048179. doi: 10.1136/bmjopen-2020-048179 (PMC8375767; doi:10.1136/bmjopen-2020-048179)
Supplement: Supplementary data [file bmjopen-2020-048179supp001.pdf]

Database: Medline 1946-date (includes epub ahead of print and in process citations. Updated daily)
Search Strategy:
----
1 Dialysis/ or dialysis.mp. (171204)
2 dialyses.mp. (821)
3 dialysate.mp. or Dialysis Solutions/ (13351)
4 dialysates.mp. (1416)
5 dialyzate.mp. (165)
6 dialyzates.mp. (28)
7 microdialysis.mp. or Microdialysis/ (17875)
8 microdialyses.mp. (4)
9 kidney replacement therapy.mp. (301)
10 kidney replacement therapies.mp. (27)
11 renal replacement therapy.mp. or Renal Replacement Therapy/ (15443)
12 renal replacement therapies.mp. (821)
13 artificial kidney*.mp. or Kidneys, Artificial/ (4751)
14 blood dialyser*.mp. (4)
15 blood dialyzer*.mp. (22)
16 Renal Dialysis/ (92294)
17 CAPD.mp. or Peritoneal Dialysis, Continuous Ambulatory/ (11179)
18 *dialysis/ (2279)
19 *dialyses/ (2279)
20 Hemofiltration/ or hemofiltration*.mp. (6341)
21 haemofiltration*.mp. (961)
22 ultrafilteration*.mp. (5)
23 biofilteration*.mp. (2)
24 1 or 2 or 3 or 4 or 5 or 6 or 7 or 8 or 9 or 10 or 11 or 12 or 13 or 14 or 15 or 16 or 17 or 18 or 19 or 20 or 21 or 22 or 23 (202167)
25 quality of life.mp. or "Quality of Life"/ (357611)
26 Qol.mp. (40312)
27 Hql.mp. (99)
28 Hqol.mp. (110)
29 H qol.mp. (8)
30 Hrqol.mp. (17377)
31 Hr qol.mp. (827)
32 life quality.mp. (7693)
33 value of life.mp. or "Value of Life"/ (6115)
34 quality adjusted life year*.mp. or Quality-Adjusted Life Years/ (18974)
35 QALY.mp. (9183)
36 Disability adjusted life year*.mp. (3584)
37 DALY.mp. (1935)
38 kidney disease quality of life-SF.mp. (5)
39 kidney disease quality of life.mp. (394)
40 kidney disease quality of life-36.mp. (47)
41 KDQOL*.mp. (350)
42 sf6.mp. (1658)
43 sf 6.mp. (476)
44 short form 6.mp. (111)
45 sf six.mp. (5)
46 short form six.mp. (56)
47 6-item short form.mp. (31)
48 six item short form.mp. (47)
49 sf12.mp. (410)
50 sf 12.mp. (4650)
51 short form 12.mp. (2187)

52 shortform 12.mp. (1)
53 sf twelve.mp. (1)
54 12-item short form.mp. (1245)
55 sf16.mp. (8)
56 sf 16.mp. (21)
57 short form 16.mp. (4)
58 sf sixteen.mp. (1)
59 short form sixteen.mp. (1)
60 16-item short form.mp. (13)
61 sf20.mp. (39)
62 sf 20.mp. (298)
63 short form 20.mp. (83)
64 sf twenty.mp. (7)
65 short form twenty.mp. (1)
66 20-item short form.mp. (31)
67 sf36.mp. (1313)
68 sf 36.mp. (21025)
69 short form 36.mp. (10446)
70 shortform 36.mp. (13)
71 sf thirty six.mp. (1)
72 short form thirty six.mp. (1)
73 36-item short form.mp. (4489)
74 SF-36.mp. (21025)
75 SF-6D.mp. (798)
76 SF-6.mp. (476)
77 SF-12.mp. (4650)
78 SF-20.mp. (298)
79 SF-16.mp. (21)
80 25 or 26 or 27 or 28 or 29 or 30 or 31 or 32 or 33 or 34 or 35 or 36 or 37 or 38 or 39 or 40 or 41 or 42 or 43 or 44 or 45 or 46 or 47 or 48 or 49 or 50 or 51 or 52 or 53 or 54 or 55 or 56 or 57 or 58 or 59 or 60 or 61 or 62 or 63 or 64 or 65 or 66 or 67 or 68 or 69 or 70 or 71 or 72 or 73 or 74 or 75 or 76 or 77 or 78 or 79 (382375)
81 24 and 80 (6724)
82 Randomized Controlled Trials as Topic/ (138501)
83 Clinical Trials as Topic/ (193802)
84 trial*.mp. (1756963)
85 clinical trial.mp. or Clinical Trial/ (721882)
86 randomi?ed control* trial.mp. (560831)
87 non randomised study.mp. (411)
88 randomised study.mp. (4303)
89 non randomized study.mp. (1307)
90 randomized study.mp. (26638)
91 82 or 83 or 84 or 85 or 86 or 87 or 88 or 89 or 90 (1762534)
92 81 and 91 (1251)

N=1,251
