# Supplementary material for: Measuring quality of life in trials including patients on dialysis: how are transplants and mortality incorporated into the analysis? A systematic review protocol
Source: BMJ Open. 2021 Aug 18;11(8):e048179. doi: 10.1136/bmjopen-2020-048179 (PMC8375767; doi:10.1136/bmjopen-2020-048179)
Supplement: Author's manuscript [file bmjopen-2020-048179.draft_revisions.pdf]

BMJ Open is committed to open peer review. As part of this commitment we make the peer review history of every article we publish publicly available.

When an article is published we post the peer reviewers' comments and the authors' responses online. We also post the versions of the paper that were used during peer review. These are the versions that the peer review comments apply to.

The versions of the paper that follow are the versions that were submitted during the peer review process. They are not the versions of record or the final published versions. They should not be cited or distributed as the published version of this manuscript.

BMJ Open is an open access journal and the full, final, typeset and author-corrected version of record of the manuscript is available on our site with no access controls, subscription charges or pay-per-view fees (http://bmjopen.bmj.com).

If you have any questions on BMJ Open's open peer review process please email info.bmjopen@bmj.com

BMJ Open

Measuring quality of life in trials including patients on dialysis: How are transplants and mortality incorporated into the analysis? A systematic review protocol

Journal:	*BMJ Open*
Manuscript ID	bmjopen-2020-048179
Article Type:	Protocol
Date Submitted by the Author:	21-Dec-2020

Complete List of Authors:	Worboys, Hannah; University of Leicester, Department of Health Sciences<br>Gray, Laura; University of Leicester, Department of Health Sciences<br>Cooper, Nicola; University of Leicester, Department of Health Sciences<br>Burton, James; University of Leicester, Department of Cardiovascular Sciences

Keywords:	Dialysis < NEPHROLOGY, Chronic renal failure < NEPHROLOGY, NEPHROLOGY

SCHOLARONE™
Manuscripts

I, the Submitting Author has the right to grant and does grant on behalf of all authors of the Work (as defined in the below author licence), an exclusive licence and/or a non-exclusive licence for contributions from authors who are: i) UK Crown employees; ii) where BMJ has agreed a CC-BY licence shall apply, and/or iii) in accordance with the terms applicable for US Federal Government officers or employees acting as part of their official duties; on a worldwide, perpetual, irrevocable, royalty-free basis to BMJ Publishing Group Ltd ("BMJ") its licensees and where the relevant Journal is co-owned by BMJ to the co-owners of the Journal, to publish the Work in this journal and any other BMJ products and to exploit all rights, as set out in our licence.

The Submitting Author accepts and understands that any supply made under these terms is made by BMJ to the Submitting Author unless you are acting as an employee on behalf of your employer or a postgraduate student of an affiliated institution which is paying any applicable article publishing charge ("APC") for Open Access articles. Where the Submitting Author wishes to make the Work available on an Open Access basis (and intends to pay the relevant APC), the terms of reuse of such Open Access shall be governed by a Creative Commons licence – details of these licences and which Creative Commons licence will apply to this Work are set out in our licence referred to above.

Other than as permitted in any relevant BMJ Author's Self Archiving Policies, I confirm this Work has not been accepted for publication elsewhere, is not being considered for publication elsewhere and does not duplicate material already published. I confirm all authors consent to publication of this Work and authorise the granting of this licence.

For peer review only

Measuring quality of life in trials including patients on dialysis: How are transplants and mortality incorporated into the analysis? A systematic review protocol

Hannah Worboys[1], Laura J Gray[1], Nicola J Cooper[1], James O Burton[2],

1. Department of Health Sciences, University of Leicester, Leicester, UK.
2. Department of Cardiovascular Sciences, University of Leicester, Leicester, UK.

Correspondence to: Hannah Worboys; hw315@leicester.ac.uk

Key words: Chronic Kidney Disease, Dialysis, Quality of Life

Word count: 2,515

For peer review only

ABSTRACT

Introduction

It is estimated that 25,000 people in the UK receive dialysis. Dialysis is an intrusive and time-consuming intervention that causes significant reductions in quality of life. When enrolled in a clinical trial, often some patients are lost prior to follow-up either because they die or receive a kidney transplant. It is unclear how these patients are dealt with in the analysis of quality of life measures within clinical trials. This review will assess current practice for dealing with transplants and mortality in trials of patients on dialysis.

Methods and Analysis

A systematic search of electronic databases, including Medline and the Cochrane Library, will be conducted to find clinical trials enrolling patients on dialysis. Trials included will measure quality of life using either the KDQoL or SF-12 (any variation). Ongoing trials will be identified through a search of trial registers. Articles will be screened against inclusion/exclusion criteria. Data will be extracted using a predetermined data extraction form. General information such as the title, location and the trial design will be extracted as well as information regarding the methods used to deal with patients that died or received a transplant before follow up. Two independent reviewers will perform screening, extraction and risk of bias. Disagreements will be resolved by discussion or by a third reviewer. Data synthesis will be performed as a narrative summary.

Ethics and Dissemination

Ethics approval is not required. Dissemination will be by publication in a peer-reviewed journal.

PROSPERO Registration Number

CRD42020223869

ARTICLE SUMMARY

Strengths and Limitations of this Study

- This review aims to collate the methods employed for dealing with specific missing outcome data, patients who receive a transplant or die prior to follow up.
- The Preferred Reported Items for Systematic Reviews and Meta-Analyses (PRISMA) guidelines will be adhered to.

For peer review only

For peer review only - http://bmjopen.bmj.com/site/about/guidelines.xhtml

- A comprehensive search strategy, peer-reviewed by a clinician, librarian and methodologists, will be undertaken in several electronic databases of peer-reviewed journals and trial registers.
- It is acknowledged that CKD has high prevalence across the world; however, including only English language publications is not expected to have a significant effect on the results of this review.
- It is expected that many studies will have included insufficient information about the conduct of their data analysis; authors will be contacted, which may delay the review but will help resolve this issue.

INTRODUCTION

The number of patients suffering from chronic kidney disease (CKD) across the world is increasing. Advanced cases of CKD require dialysis to replace the function of the kidneys. It is approximated that there are 25,000 dialysis patients in the UK[1]. Dialysis is costly and requires many health care resources, including hospital equipment, nurse and physician time. It is also an intrusive procedure and requires multiple trips to a clinic every week. This imposes a significant burden on patients and their families. Patients are often limited in their ability to work and care for dependents, as well as experiencing headaches, fatigue and other side effects as a result of dialysis [2]. These factors significantly affect both adherence to the treatment and quality of life (QoL). On average, one in three patients with CKD experiences depression [3] and non-adherence to treatment is escalating levels of patient mortality[4]. The adverse effects on the wellbeing of patients with CKD are well known; this has led to a large increase in clinical trials that aim to improve QoL. Despite a large number of trials, QoL amongst patients on dialysis remains low. Many trials have failed to reflect important improvements in outcomes and hence have not lead to changes in practice. This may be linked to limitations of the trial design such as small sample size, inability to blind participants, using QoL measures not validated in the dialysis patient population and dealing with missing data inadequately [5].

The problems relating to recruitment and retention to dialysis trials are well-documented in the literature. Strategies to improve recruitment into kidney disease trials have been developed and include improving education, increasing information prior to potential trials and incentivising participation[6]. As well as this, core outcome sets such as those developed by the Standardised Outcomes in Nephrology for

For peer review only - http://bmjopen.bmj.com/site/about/guidelines.xhtml

Haemodialysis[7] (SONG-HD) Collaboration try to ensure that the most appropriate outcomes are incorporated into trial design. Initiatives such as SONG-HD has led to the recognition of the importance for condition specific patient reported outcome measures (PROMs). The kidney disease quality of life (KDQoL) questionnaire is a condition-specific QoL measure that is designed to measure health-related QoL issues associated with CKD [8]. The KDQoL-36 is a short form of the KDQoL that includes the SF-12, as well as questions relating to; the burden of kidney disease; symptoms and problems of kidney disease; and the effects of kidney disease. The KDQoL-36 scores highly in consistency, validity and reliability [9] and is a recommended PROM for use in dialysis patients. The KDQoL is used globally and has been translated into over 20 languages [10]. The SF-12 is a generic measure of health that has also been used widely in trials measuring QoL amongst CKD patients. It is anticipated that the KDQoL and SF-12 are the most common QoL measures used in trials of dialysis patients.

Despite many researchers developing appropriate ways to deal with missing data, little is mentioned about the specific types of missing data that occur in dialysis trials. During trials in dialysis, a number of patients will either receive a kidney transplant or die before the intended data collection point. For example, the PIVOTAL trial[11], had 515 deaths (24% of patients randomised) and 371 transplants (17% of patients randomised) and had a median follow-up of 2.1 years. Outcomes for patients who have a transplant may or may not be collected. For those who have died, outcomes may be set to an arbitrary value or to missing in the analysis. Therefore these two events may result in missing values. It is unclear how this missing data has been dealt with in the data analysis of previous trials. Intention to treat (ITT) analysis is the gold standard for randomised controlled trials (RCTs) and involves analysing all patients that were randomised into a trial, irrespective of the subsequent events. ITT is the preferred approach as it reduces the risk of bias, maintains randomisation and better reflects the reality of receiving treatment in the real world. Under ITT, it is therefore inappropriate to exclude missing outcome data that occurs due to patients who receive a transplant or die prior to follow up. Excluding these patients may lead to over/underestimating the intervention effect. It is important to find an appropriate method to account for these two events, such that an accurate estimation of the intervention effect can take place.

For peer review only - http://bmjopen.bmj.com/site/about/guidelines.xhtml

Objectives

This systematic review aims to evaluate the current practice of dealing with the missing outcome data as a result of patient mortality or kidney transplantation. The review will appraise trials that use any variations of the KDQoL or SF-12 when measuring QoL.

The proposed systematic review will answer the following questions.

1. Do trials of dialysis patients that measure the QoL report how they deal with patients who receive a transplant or die in the analysis?
2. What methods are currently used for dealing with death/transplant in the analysis of dialysis trials?

METHODS

The systematic review will follow the Preferred Reporting Items for Systematic Reviews and Meta-Analyses (PRISMA) guidelines for reporting the results of systematic reviews [12]. The PRISMA-P protocol checklist for systematic review protocols was used when writing this protocol [13] and is provided in the supplementary information.

Peer-reviewed Literature

The search strategy will be developed by the primary author with the assistance of a specialist Health Sciences Librarian and peer-reviewed by an Honorary Consultant Nephrologist at the Leicester General Hospital. The electronic databases Medline, Web of Science, Cochrane Central Register of Controlled Trials (CENTRAL), Scopus and Cumulative Index of Nursing and Allied Health Literature (CINHAL) will be searched using combinations of keywords and topics. The main search strategy, written for Medline, is included in the supplementary information and will be adapted for use in subsequent databases. Full search strategies will be included in the Appendix of the review. The databases will be searched from inception to 31/12/2020. Searches will be re-run before the final analysis; any studies published in the intervening period will be retrieved and included. The search will be limited to publications readily available in the English language. Search results will be exported and sorted using EndNote X9. All studies included will have their reference lists checked for additional studies.

Ongoing Studies

For peer review only - http://bmjopen.bmj.com/site/about/guidelines.xhtml

Trials registers are an important source for identifying additional randomised trials. ClinicalTrials.gov, the International Clinical Trials Register Search Portal (ICTRP), the European Union Clinical Trials Register (EUDRaCT) and the International Standard Randomised Controlled Trial Number (ISRCTN) will be searched to identify ongoing studies for inclusion in the review. Thorough searches will reduce the risk of publication bias.

Inclusion/Exclusion Criteria

For inclusion, the trial must be testing an intervention within a population of dialysis patients. The trial must measure QoL as an outcome. Trials must use the KDQoL (any variation) or the SF-12 (any variation) to measure QoL. Trials considered for inclusion will be phase 3 clinical trials of any design. Trials included will have recruited adult populations (18+) as the KDQoL and SF-12 are unlikely to be asked directly to children. There is no restriction on the setting or location of the trial. Articles must be either reported or readily available in the English language.

Screening

Titles, authors, abstracts, the date and location of publication will be exported into EndNote X9. Duplicates will be removed. Titles and abstracts will be screened for inclusion. The full-text articles will be screened for confirmation of suitability if the abstracts do not have sufficient information. Authors will be contacted if after full-text screening it is still not clear whether to include/exclude an article. All articles will be screened independently by two reviewers. Any disagreement on inclusion will be resolved by discussion or with another reviewer. A characteristics of excluded studies table will be presented in the Appendix of the review, detailing reasons for exclusion.

Data extraction

After screening, data extraction will be performed using a predetermined extraction form. Two data extraction forms will exist; one focusing on the approach used in completed trials; the other focusing on the planned approach for ongoing trials. Two reviewers will do the extraction, with differences discussed. A third reviewer will resolve if necessary. Pilot data extraction will test the forms prior to the main analysis.

For peer review only - http://bmjopen.bmj.com/site/about/guidelines.xhtml

Data to be extracted for completed and ongoing trials:

- Title
- Author(s)
- Correspondence
- Study design
- Study location
- Intervention
- Control
- Primary outcome
- QoL measure
- Length of follow-up

Additional information for completed trials

- Date(s) of patient recruitment
- Publication type
- Publication date
- Country of publication
- Journal of publication
- Approach to main analysis (intention to treat, complete case, per protocol)
- Number of patients randomised
- Death mentioned (Y/N)
- Transplant mentioned (Y/N)
- Number of deaths
- Number of transplants
- Total number of withdrawal
- The method used to account for deaths in the analysis of QoL
- The method used to account for transplant in the analysis of QoL

Additional information for ongoing trials

- Status of trial
- Anticipated sample size
- Planned approach to main analysis (intention to treat, complete case, per protocol)
- Trial protocol available (Y/N)
- Possibility of death leading to missing outcomes mentioned (Y/N)

For peer review only - http://bmjopen.bmj.com/site/about/guidelines.xhtml

- Possibility of transplant leading to missing outcomes mentioned (Y/N)
- Method to be used to account for deaths in the analysis of QoL
- Method to be used to account for transplants in the analysis of QoL

Outcomes

- The number of studies that mention how death or transplants are dealt with in the analysis.
- Methods used to account for deaths and transplants in the analysis of QoL.
- Most common methods used.
- Number of studies that fail to mention how they account for deaths and transplants in the analysis of QoL.
- Number of studies where death or transplants account for more than 5% of patients randomised.
- Associations between methods and the risk of bias.

Risk of Bias Assessment

RCTs are the gold standard of evidence-based medicine. However, they are open to bias if poorly designed or executed. When conducting a systematic review, in order to make reliable conclusions, the reviewers must consider the quality of the studies included. The Cochrane collaboration's tool for assessing the risk of bias (RoB) [14] has been used in a significant number of previous systematic reviews. An updated version of this tool, the RoB 2.0 [15] will be used in this review to assess the RoB for each trial. Judgements about the RoB will be made independently by two reviewers and disagreements resolved through discussion.

There are five domains of bias, each with several elements. Each domain is concluded as "low", "high" or "some concerning" level of risk. Completion of all domains leads to an overall RoB judgement. The aim of the quality assessment is not to further exclude studies from the review but instead to find the strengths and limitations of each study.

The sources of bias to be assessed are

- Domain 1: Risk of bias arising from the randomisation process.
- Domain 2: Risk of bias due to deviations from the intended interventions.
- Domain 3. Risk of bias due to missing outcome data.
- Domain 4: Risk of bias in measurement of the outcome.

For peer review only - http://bmjopen.bmj.com/site/about/guidelines.xhtml

- Domain 5: Risk of bias in the selection of the reported result.

For non-randomised controlled trials, the Cochrane RoB for non-randomised studies (ROBINS-I) [16] will be used to assess the quality of the trial. Similar to the RoB tool, the ROBINS-I will lead to an overall RoB judgement for each trial.

Data Synthesis

Completed trials

Completed trials that meet the inclusion criteria will be analysed using either RoB or ROBINS-I tool. The key information from the extracted data, alongside the RoB will be tabulated. Articles will be grouped into whether they have stated the methods used to deal with death and transplant patients. Articles that have will then be categorised into methods used. Authors of articles that have not will be contacted for this information. Responses will then be categorised into methods used or non-response. The results will be synthesised using descriptive statistics. Where possible, the percentage of patients who die or receive a transplant out of the total number of patients randomised will be calculated respectively for each trial. This review will highlight the common methods as well as the percentage of studies that fail to mention this issue. Associations between reporting and the RoB will be assessed. The results of the review will be interpreted in line with the limitations of each study.

Ongoing trials

The key information from the extracted data will be tabulated. Articles will be grouped into whether they have anticipated the methods they will use to deal with death and transplant patients. Articles that have will then be categorised into methods to be used. Authors of articles that have not will be contacted for this information.

Extensive systematic searches will strengthen the findings of this review. It is anticipated that this review will identify the methods used in current practice are inconsistent and inadequate. The results from this review will highlight the importance of using an acceptable method when dealing with this issue in nephrology trials.

For peer review only - http://bmjopen.bmj.com/site/about/guidelines.xhtml

Ethics and Dissemination

No ethical approval is needed as all data used is available publicly. A summary of the extracted data will be available within the final publication. After completion, expected to be no later than 03/01/2022, the review will be submitted to a peer-reviewed journal.

Author Contributions: HW, LJG, NJC and JB were involved in all stages of writing the systematic review protocol and contributed to the concept and design, drafting, revision and approval of the final version. The search strategy for peer-reviewed literature and trial registers was created by HW and reviewed by JB and LJG.

Funding award: NIHR Applied Research Collaboration East Midlands

Funding: HW is funded by the National Institute for Health Research (NIHR) Applied Research Collaboration East Midlands (ARC EM). The views expressed are those of the author(s) and not necessarily those of the NIHR or the Department of Health and Social Care.

Data statement: A summary of the extracted data will be available within the final publication.

Patient and public involvement: It was not appropriate or possible to involve patients or the public in the design, or conduct, or reporting, or dissemination plans of our research.

Competing interests: None

ORCID IDs http://orcid.org/0000-0003-3958-0180

For peer review only

References

1. UK Renal Registry. UK Renal Registry 22nd Annual Report - data to 31/12/2018. Bristol, UK, 2020.
2. Find Specialist. Hemodialysis – NephCure Kidney International 2020 [Available from: https://nephcure.org/hemodialysis accessed 12/11/2020.
3. Kidney Care UK. Facts and Stats 2020 [Available from: https://www.kidneycareuk.org/news-and-campaigns/facts-and-stats/ accessed 26 October 2020.
4. Clarke AL, Yates T, Smith AC, et al. Patient's perceptions of chronic kidney disease and their association with psychosocial and clinical outcomes: a narrative review. *Clinical Kidney Journal* 2016;9((3)):494-502.
5. Novak JE, Inrig JK, Patel UD, et al. Negative trials in nephrology: what can we learn? *Kidney international* 2008;74(9):1121-27. doi: 10.1038/ki.2008.286
6. Baigent C, Herrington WG, Coresh J, et al. Challenges in conducting clinical trials in nephrology: conclusions from a Kidney Disease—Improving Global Outcomes (KDIGO) Controversies Conference. *Kidney international* 2017;92(2):297-305. doi: 10.1016/j.kint.2017.04.019
7. Tong A, Manns B, Hemmelgarn B, et al. Establishing core outcome domains in hemodialysis: report of the Standardized Outcomes in Nephrology–Hemodialysis (SONG-HD) consensus workshop. *American Journal of Kidney Diseases* 2017;69(1):97-107.

For peer review only - http://bmjopen.bmj.com/site/about/guidelines.xhtml

8. Hays RD, Kallich JD, Mapes DL, et al. Development of the Kidney Disease Quality of Life (KDQOLTM) Instrument. *Quality of life research* 1994;3(5):329-38. doi: 10.1007/bf00451725
9. Aiyegbusi OL, Kyte D, Cockwell P, et al. Measurement properties of patient-reported outcome measures (PROMs) used in adult patients with chronic kidney disease: A systematic review. *PloS one* 2017;12(6):e0179733-e33. doi: 10.1371/journal.pone.0179733
10. RAND. Kidney Disease Quality of Life Instrument 2020 [Available from: https://www.rand.org/health-care/surveys_tools/kdqol.html accessed 29 October 2020.
11. Macdougall IC, White C, Anker SD, et al. Intravenous iron in patients undergoing maintenance hemodialysis. *New England Journal of Medicine* 2019;380(5):447-58.
12. Moher D, Liberati A, Tetzlaff J, et al. Preferred reporting items for systematic reviews and meta-analyses: the PRISMA statement. *BMJ* 2009;339(jul21 1):b2535-b35. doi: 10.1136/bmj.b2535
13. Shamseer L, Moher D, Clarke M, et al. Preferred reporting items for systematic review and meta-analysis protocols (PRISMA-P) 2015: elaboration and explanation. *BMJ : British Medical Journal* 2015;349(jan02 1):g7647-g47. doi: 10.1136/bmj.g7647
14. Higgins JP, Altman DG, Gøtzsche PC, et al. The Cochrane Collaboration's tool for assessing risk of bias in randomised trials. *Bmj* 2011;343:d5928.
15. Sterne JAC, Savović J, Page MJ, et al. RoB 2: a revised tool for assessing risk of bias in randomised trials. *BMJ* 2019;366:l4898-l98. doi: 10.1136/bmj.l4898
16. Sterne JAC, Hernán MA, Reeves BC, et al. ROBINS-I: a tool for assessing risk of bias in non-randomised studies of interventions. *BMJ* 2016;355:i4919-i19. doi: 10.1136/bmj.i4919

For peer review only

For peer review only - http://bmjopen.bmj.com/site/about/guidelines.xhtml

Database: Medline 1946-date (includes epub ahead of print and in process citations. Updated daily)

Search Strategy:

----

1 Dialysis/ or dialysis.mp. (171204)

2 dialyses.mp. (821)

3 dialysate.mp. or Dialysis Solutions/ (13351)

4 dialysates.mp. (1416)

5 dialyzate.mp. (165)

6 dialyzates.mp. (28)

7 microdialysis.mp. or Microdialysis/ (17875)

8 microdialyses.mp. (4)

9 kidney replacement therapy.mp. (301)

10 kidney replacement therapies.mp. (27)

11 renal replacement therapy.mp. or Renal Replacement Therapy/ (15443)

12 renal replacement therapies.mp. (821)

13 artificial kidney*.mp. or Kidneys, Artificial/ (4751)

14 blood dialyser*.mp. (4)

15 blood dialyzer*.mp. (22)

16 Renal Dialysis/ (92294)

17 CAPD.mp. or Peritoneal Dialysis, Continuous Ambulatory/ (11179)

18 *dialysis/ (2279)

19 *dialyses/ (2279)

20 Hemofiltration/ or hemofiltration*.mp. (6341)

21 haemofiltration*.mp. (961)

22 ultrafilteration*.mp. (5)

23 biofilteration*.mp. (2)

24 1 or 2 or 3 or 4 or 5 or 6 or 7 or 8 or 9 or 10 or 11 or 12 or 13 or 14 or 15 or 16 or 17 or 18 or 19 or 20 or 21 or 22 or 23 (202167)

25 quality of life.mp. or "Quality of Life"/ (357611)

26 Qol.mp. (40312)

27 Hql.mp. (99)

28 Hqol.mp. (110)

29 H qol.mp. (8)

30 Hrqol.mp. (17377)

31 Hr qol.mp. (827)

32 life quality.mp. (7693)

For peer review only

33 value of life.mp. or "Value of Life"/ (6115)
34 quality adjusted life year*.mp. or Quality-Adjusted Life Years/ (18974)
35 QALY.mp. (9183)
36 Disability adjusted life year*.mp. (3584)
37 DALY.mp. (1935)
38 kidney disease quality of life-SF.mp. (5)
39 kidney disease quality of life.mp. (394)
40 kidney disease quality of life-36.mp. (47)
41 KDQOL*.mp. (350)
42 sf6.mp. (1658)
43 sf 6.mp. (476)
44 short form 6.mp. (111)
45 sf six.mp. (5)
46 short form six.mp. (56)
47 6-item short form.mp. (31)
48 six item short form.mp. (47)
49 sf12.mp. (410)
50 sf 12.mp. (4650)
51 short form 12.mp. (2187)
52 shortform 12.mp. (1)
53 sf twelve.mp. (1)
54 12-item short form.mp. (1245)
55 sf16.mp. (8)
56 sf 16.mp. (21)
57 short form 16.mp. (4)
58 sf sixteen.mp. (1)
59 short form sixteen.mp. (1)
60 16-item short form.mp. (13)
61 sf20.mp. (39)
62 sf 20.mp. (298)
63 short form 20.mp. (83)
64 sf twenty.mp. (7)
65 short form twenty.mp. (1)
66 20-item short form.mp. (31)
67 sf36.mp. (1313)
68 sf 36.mp. (21025)
69 short form 36.mp. (10446)

For peer review only

70 shortform 36.mp. (13)
71 sf thirty six.mp. (1)
72 short form thirty six.mp. (1)
73 36-item short form.mp. (4489)
74 SF-36.mp. (21025)
75 SF-6D.mp. (798)
76 SF-6.mp. (476)
77 SF-12.mp. (4650)
78 SF-20.mp. (298)
79 SF-16.mp. (21)
80 25 or 26 or 27 or 28 or 29 or 30 or 31 or 32 or 33 or 34 or 35 or 36 or 37 or 38 or 39 or 40 or 41 or 42 or 43 or 44 or 45 or 46 or 47 or 48 or 49 or 50 or 51 or 52 or 53 or 54 or 55 or 56 or 57 or 58 or 59 or 60 or 61 or 62 or 63 or 64 or 65 or 66 or 67 or 68 or 69 or 70 or 71 or 72 or 73 or 74 or 75 or 76 or 77 or 78 or 79 (382375)
81 24 and 80 (6724)
82 Randomized Controlled Trials as Topic/ (138501)
83 Clinical Trials as Topic/ (193802)
84 trial*.mp. (1756963)
85 clinical trial.mp. or Clinical Trial/ (721882)
86 randomi?ed control* trial.mp. (560831)
87 non randomised study.mp. (411)
88 randomised study.mp. (4303)
89 non randomized study.mp. (1307)
90 randomized study.mp. (26638)
91 82 or 83 or 84 or 85 or 86 or 87 or 88 or 89 or 90 (1762534)
92 81 and 91 (1251)

N=1,251

For peer review only

Reporting checklist for protocol of a systematic review.

Based on the PRISMA-P guidelines.

Instructions to authors

Complete this checklist by entering the page numbers from your manuscript where readers will find each of the items listed below.

Your article may not currently address all the items on the checklist. Please modify your text to include the missing information. If you are certain that an item does not apply, please write "n/a" and provide a short explanation.

Upload your completed checklist as an extra file when you submit to a journal.

In your methods section, say that you used the PRISMA-Preporting guidelines, and cite them as:

Moher D, Shamseer L, Clarke M, Ghersi D, Liberati A, Petticrew M, Shekelle P, Stewart LA. Preferred Reporting Items for Systematic Review and Meta-Analysis Protocols (PRISMA-P) 2015 statement. Syst Rev. 2015;4(1):1.

For peer review only

		Reporting Item	Page Number
**Title**			
Identification	#1a	Identify the report as a protocol of a systematic review	1
Update	#1b	If the protocol is for an update of a previous systematic review, identify as such	n/a

**Registration**			
	#2	If registered, provide the name of the registry (such as PROSPERO) and registration number	2
**Authors**			
Contact	#3a	Provide name, institutional affiliation, e-mail address of all protocol authors; provide physical mailing address of corresponding author	2
Contribution	#3b	Describe contributions of protocol authors and identify the guarantor of the review	7
**Amendments**			
	#4	If the protocol represents an amendment of a previously completed or published protocol, identify as such and list changes; otherwise, state plan for documenting important protocol amendments	n/a
**Support**			
Sources	#5a	Indicate sources of financial or other support for the review	7
Sponsor	#5b	Provide name for the review funder and / or sponsor	7
Role of sponsor or funder	#5c	Describe roles of funder(s), sponsor(s), and / or institution(s), if any, in developing the protocol	7
**Introduction**			

For peer review only

Rationale	#6	Describe the rationale for the review in the context of what is already known	3/4
Objectives	#7	Provide an explicit statement of the question(s) the review will address with reference to participants, interventions, comparators, and outcomes (PICO)	4
**Methods**			
Eligibility criteria	#8	Specify the study characteristics (such as PICO, study design, setting, time frame) and report characteristics (such as years considered, language, publication status) to be used as criteria for eligibility for the review	5
Information sources	#9	Describe all intended information sources (such as electronic databases, contact with study authors, trial registers or other grey literature sources) with planned dates of coverage	4/5
Search strategy	#10	Present draft of search strategy to be used for at least one electronic database, including planned limits, such that it could be repeated	9
Study records - data management	#11a	Describe the mechanism(s) that will be used to manage records and data throughout the review	5/6
Study records - selection process	#11b	State the process that will be used for selecting studies (such as two independent reviewers) through each phase of the review (that is, screening, eligibility and inclusion in meta-analysis)	5

For peer review only

Study records - data collection process	#11c	Describe planned method of extracting data from reports (such as piloting forms, done independently, in duplicate), any processes for obtaining and confirming data from investigators	5/6
Data items	#12	List and define all variables for which data will be sought (such as PICO items, funding sources), any pre-planned data assumptions and simplifications	5/6
Outcomes and prioritization	#13	List and define all outcomes for which data will be sought, including prioritization of main and additional outcomes, with rationale	6
Risk of bias in individual studies	#14	Describe anticipated methods for assessing risk of bias of individual studies, including whether this will be done at the outcome or study level, or both; state how this information will be used in data synthesis	5
Data synthesis	#15a	Describe criteria under which study data will be quantitatively synthesised	n/a
Data synthesis	#15b	If data are appropriate for quantitative synthesis, describe planned summary measures, methods of handling data and methods of combining data from studies, including any planned exploration of consistency (such as I2, Kendall's τ)	n/a
Data synthesis	#15c	Describe any proposed additional analyses (such as sensitivity or subgroup analyses, meta-regression)	n/a

For peer review only

Data synthesis	#15d	If quantitative synthesis is not appropriate, describe the type of summary planned	7
Meta-bias(es)	#16	Specify any planned assessment of meta-bias(es) (such as publication bias across studies, selective reporting within studies)	n/a
Confidence in cumulative evidence	#17	Describe how the strength of the body of evidence will be assessed (such as GRADE)	7

The PRISMA-P checklist is distributed under the terms of the Creative Commons Attribution License CC-BY 4.0. This checklist was completed on 04. December 2020 using https://www.goodreports.org/, a tool made by the EQUATOR Network in collaboration with Penelope.ai

For peer review only

PRISMA-P Checklist with information

SECTION AND TOPIC	ITEM NO	INFORMATION
**ADMINISTRATIVE INFORMATION**		
Title:		
Identification	1a	Measuring quality of life in trials including patients on dialysis: How are transplants and mortality incorporated into the analysis? A systematic review protocol
Update	1b	N/A
Registration	2	CRD42020223869
Authors:		
Contact	3a	➢ Hannah Worboys, hw315@leicester.ac.uk, Department of Health Sciences, University of Leicester, Leicester, UK.<br>➢ Professor Laura Gray, lg48@leicester.ac.uk, Department of Health Sciences, University of Leicester, Leicester, UK.<br>➢ Professor Nicola Cooper, njc21@leicester.ac.uk, Department of Health Sciences, University of Leicester, Leicester, UK.<br>➢ Professor James Burton, jb343@leicester.ac.uk, Department of Cardiovascular Sciences, University of Leicester, UK.
Contributions	3b	HW, LJG and NJC were involved in all stages of writing the systematic review protocol and contributed to the concept and design, drafting, revision and approval of the final version. The search strategy for peer-reviewed literature and trial registers was created by HW.
Amendments	4	N/A
Support:		
Sources	5a	This systematic review protocol is part of a PhD project being undertaken by HW and funded by the NIHR ARC EM.
Sponsor	5b	N/A
Role of sponsor or funder	5c	The funder had no role in the development of this protocol
**INTRODUCTION**		
Rationale	6	It is estimated that 25,000 people in the UK receive dialysis. Dialysis is an intrusive and time-consuming process that often causes significant reductions in quality of life. When enrolled in a clinical trial, it is estimated that 10% of patients will either die or receive a transplant within six months. It is unclear how these patients are dealt with in the analysis of quality of life measures. This review will assess current practice for dealing with transplants and mortality in trials of patients on dialysis.

For peer review only

Objectives	7	➢ Do trials of dialysis patients that measure the QoL report how they deal with patients who receive a transplant or die in the analysis?<br>➢ What methods are currently used for dealing with death/transplant in the analysis of dialysis trials?
**METHODS**		
Eligibility criteria	8	Phase 3 clinical trials of any design<br>Adult population, diagnosed with CKD, enrolled on dialysis<br>Incorporate measuring quality of life through use of the KDQoL or SF12
Information sources	9	**Peer-reviewed literature**<br>Medline, Web of Science , CENTRAL, CINAHL<br>Search databases from inception to 31/12/2020<br>Searched will be reran just before analysis<br>Limited to publications readily available in the English language<br>**Trial registers**<br>ClinicalTrials.gov, ICTRP, EUDRaCT, ISRCTN
Search strategy	10	Present draft of search strategy to be used for at least one electronic database, including planned limits, such that it could be repeated
Study records:		
Data management	11a	EndNote X9 will be used to store and manage and de-duplicate the references resulting from the searches and also used in the screening of titles and abstract process.
Selection process	11b	The reviewers (HW and GW) will independently screen the full texts of those studies and reports identified as having inclusion potential against the inclusion/exclusion criteria. Each stage of the screen process will be recorded using EndNote X9. Disagreement between the reviewers at all stages of screening is to be resolved by discussion and, if necessary, by the consultation of a third reviewer.
Data collection process	11c	Data extraction will be performed independently by two reviewers (HW and GW) using a pre-tested extraction form. Any missing information will be sought by contacting the authors. Disagreement between the reviewers at all stages of screening and data extraction is to be resolved by discussion and, if necessary, by the consultation of a third reviewer
Data items	12	General<br>➢ Author<br>➢ Trial name<br>➢ Publication date and location<br>➢ Intervention and control<br>➢ Trial design

For peer review only

		➢ Trial date and location<br>➢ Approach to the main analysis<br>➢ Sample size<br>➢ Primary outcome<br>➢ QoL measure used (KDQoL or RAND short form)<br>➢ Follow-up period<br>Specific information relating to death/transplants<br>➢ Death mentioned (Y/N)<br>➢ Transplant mentioned (Y/N)<br>➢ Number of deaths stated<br>➢ Number of transplants stated<br>➢ The method used to account for deaths in the analysis of QoL<br>➢ The method used to account for transplant in the analysis of QoL
Outcomes and prioritization	13	➢ Different methods used to account for deaths and transplants in the analysis of quality of life.<br>➢ Most common methods used<br>➢ Number of studies that fail to mention how they account for deaths and transplants in the analysis of quality of life
Risk of bias in individual studies	14	RoB 2.0 for randomised trials<br>ROBINS-I for non-randomised trials
Data synthesis	15a	n/a
	15b	n/a
	15c	n/a
	15d	Narrative summary of trials
Meta-bias(es)	16	N/A
Confidence in cumulative evidence	17	The results will be interpreted in line with the limitations (RoB) of the study

*** It is strongly recommended that this checklist be read in conjunction with the PRISMA-P Explanation and Elaboration (cite when available) for important clarification on the items. Amendments to a review protocol should be tracked and dated.** **The copyright for PRISMA-P (including checklist) is held by the PRISMA-P Group and is distributed under a Creative Commons Attribution Licence 4.0.**

From: Shamseer L, Moher D, Clarke M, Ghersi D, Liberati A, Petticrew M, Shekelle P, Stewart L, PRISMA-P Group. Preferred reporting items for systematic review and meta-analysis protocols (PRISMA-P) 2015: elaboration and explanation. BMJ. 2015 Jan 2;349(jan02 1):g7647.

For peer review only

BMJ Open

Measuring quality of life in trials including patients on dialysis: How are transplants and mortality incorporated into the analysis? A systematic review protocol

Journal:	*BMJ Open*
Manuscript ID	bmjopen-2020-048179.R1
Article Type:	Protocol
Date Submitted by the Author:	21-Jul-2021
Complete List of Authors:	Worboys, Hannah; University of Leicester, Department of Health Sciences<br>Cooper, Nicola; University of Leicester, Department of Health Sciences<br>Burton, James; University of Leicester, Department of Cardiovascular Sciences<br>Gray, Laura; University of Leicester, Department of Health Sciences
**Primary Subject Heading**:	Renal medicine
Secondary Subject Heading:	Renal medicine
Keywords:	Dialysis < NEPHROLOGY, Chronic renal failure < NEPHROLOGY, NEPHROLOGY

SCHOLARONE™
Manuscripts

I, the Submitting Author has the right to grant and does grant on behalf of all authors of the Work (as defined in the below author licence), an exclusive licence and/or a non-exclusive licence for contributions from authors who are: i) UK Crown employees; ii) where BMJ has agreed a CC-BY licence shall apply, and/or iii) in accordance with the terms applicable for US Federal Government officers or employees acting as part of their official duties; on a worldwide, perpetual, irrevocable, royalty-free basis to BMJ Publishing Group Ltd ("BMJ") its licensees and where the relevant Journal is co-owned by BMJ to the co-owners of the Journal, to publish the Work in this journal and any other BMJ products and to exploit all rights, as set out in our licence.

The Submitting Author accepts and understands that any supply made under these terms is made by BMJ to the Submitting Author unless you are acting as an employee on behalf of your employer or a postgraduate student of an affiliated institution which is paying any applicable article publishing charge ("APC") for Open Access articles. Where the Submitting Author wishes to make the Work available on an Open Access basis (and intends to pay the relevant APC), the terms of reuse of such Open Access shall be governed by a Creative Commons licence – details of these licences and which Creative Commons licence will apply to this Work are set out in our licence referred to above.

Other than as permitted in any relevant BMJ Author's Self Archiving Policies, I confirm this Work has not been accepted for publication elsewhere, is not being considered for publication elsewhere and does not duplicate material already published. I confirm all authors consent to publication of this Work and authorise the granting of this licence.

For peer review only

Measuring quality of life in trials including patients on dialysis: How are transplants and mortality incorporated into the analysis? A systematic review protocol

Hannah Worboys[1], Nicola J Cooper[1], James O Burton[2], Laura J Gray[1]

1. Department of Health Sciences, University of Leicester, Leicester, UK.
2. Department of Cardiovascular Sciences, University of Leicester, Leicester, UK.

Correspondence to: Hannah Worboys; hw315@leicester.ac.uk

Key words: Chronic Kidney Disease, Dialysis, Quality of Life

Word count: 2,549

For peer review only

ABSTRACT

Introduction

It is estimated that 25,000 people in the UK receive dialysis. Dialysis is an intrusive and time-consuming intervention that causes significant reductions in quality of life. When enrolled in a clinical trial, often some patients drop out of the study either because they die, receive a kidney transplant or are lost to follow up for other reasons. It is unclear how these events are dealt with when analysing quality of life measures within clinical trials. This review will assess current practice for dealing with loss to follow-up in trials including patients on haemodialysis. The methods currently used will be analysed in terms of their adequacy and will form the basis of future work assessing the most appropriate methods to employ under these circumstances. The results of this review will feed into recommendations for future nephrology trials.

Methods and Analysis

A systematic search of electronic databases including Medline and the Cochrane Library will be conducted to find clinical trials enrolling patients on haemodialysis that measure quality of life using either the KDQoL or SF-36 (or any variation of these two measures). Ongoing trials will be identified through a search of trial registers. Articles will be screened against inclusion/exclusion criteria and data will be extracted using a predetermined data extraction form. General information such as the title, location, trial design will be extracted along with more specific information on how the study dealt with patients that died or received a transplant before the end of the follow up period. Two independent reviewers will perform screening and extraction. Disagreements will be resolved by discussion or by a third independent reviewer. Data synthesis will be performed as a narrative summary.

Ethics and Dissemination

Ethics approval is not required. Dissemination will be by publication in a peer reviewed journal.

PROSPERO Registration Number

CRD42020223869

ARTICLE SUMMARY

Strengths and Limitations of this Study

- This review aims to collate the methods employed for dealing with specific missing outcome data, patients who dropout due to transplants, adverse effects and death before the end of the follow up period.
- The Preferred Reported Items for Systematic Reviews and Meta-Analyses (PRISMA) guidelines will be adhered to.
- A comprehensive search strategy, peer reviewed by a clinician, librarian and methodologists, will be undertaken in several electronic databases of peer-reviewed journals and trial registers.

For peer review only - http://bmjopen.bmj.com/site/about/guidelines.xhtml

- It is acknowledged that CKD has high prevalence across the world, however, including only English language publications is not expected to have a significant effect on the results of this review.
- It is expected that many studies will have included insufficient information about the conduct of their data analysis, authors will be contacted which may delay the review but will help resolve this issue.

INTRODUCTION

The number of patients suffering from chronic kidney disease (CKD) across the world is increasing. Advanced cases of CKD require dialysis to replace the function of the kidneys. It is approximated that there are 25,000 dialysis patients in the UK[1]. Dialysis is costly and requires many health care resources including hospital equipment, nurse and physician time. It is also an intrusive procedure and requires multiple trips to a clinic every week. This imposes a significant burden on patients and their families. Patients are often limited in their ability to work and care for dependents, as well as experiencing headaches, fatigue and other side effects as a result of dialysis [2]. These factors significantly affect both adherence to the treatment and quality of life (QoL). On average, one in three patients with CKD experiences depression [3] and non-adherence to treatment is escalating levels of patient mortality[4]. The adverse effects on the wellbeing of patients with CKD are well known; this has led to a large increase in clinical trials that aim to improve QoL. Despite a large number of trials, QoL amongst patients on dialysis remains low. Many trials have failed to reflect important improvements in outcomes and hence have not lead to changes practice. This may be linked to limitations of the trial design such as small sample size, inability to blind participants, using QoL measures not validated in the dialysis patient population and dealing with missing data inadequately [5].

The problems relating to recruitment and retention to dialysis trials are well-documented in the literature[6]. Strategies to improve recruitment into kidney disease trials have been developed and include improving education, increasing information prior to potential trials and incentivising participation[7]. As well as this, core outcome sets such as those developed by the Standardised Outcomes in Nephrology for Haemodialysis[8] (SONG-HD) Collaboration are designed to ensure the most important outcomes are incorporated in trial design. Initiatives such as SONG-HD has led to the recognition of the importance for condition-specific patient reported outcome measures (PROMs). The kidney disease quality of life (KDQoL) questionnaire is a condition-specific QoL measure that is designed to measure health-related QoL issues associated with CKD patients [9]. The KDQoL-36 is a short form of the KDQoL that includes the SF-12, as well as questions relating to; the burden of kidney disease; symptoms and problems of kidney disease; and the effects of kidney disease. The KDQoL-36 scores highly in consistency, validity and reliability [10] and is a highly used PROM amongst dialysis patients. The KDQoL is used internationally and has been translated into over 20 languages [11]. The SF-36 is a generic measure of health that has also been used widely in trials, including measuring QoL amongst CKD patients.

Despite many researchers developing appropriate ways to deal with missing data, little is mentioned about the specific types of missing data that occur in dialysis trials.

For peer review only - http://bmjopen.bmj.com/site/about/guidelines.xhtml

During trials in dialysis, a number of patients will either receive a kidney transplant or die before the intended data collection point. For example, the PIVOTAL trial[12], had 515 deaths (24% of patients randomised) and 371 transplants (17% of patients randomised) and had a median follow-up of 2.1 years. Outcomes for patients who have a transplant may or may not be collected. For those who have died, outcomes may be set to an arbitrary value or to missing in the analysis. Therefore these two events may result in missing values. It is unclear how this missing data has been dealt with in the data analysis of previous trials. Intention to treat (ITT) analysis is the gold standard for randomised controlled trials (RCTs) and involves analysing all patients that were randomised into a trial irrespective of the subsequent events. ITT is the preferred approach as it reduces the risk of bias, maintains randomisation and better reflects the reality of receiving treatment in the real world. Under ITT, it is therefore inappropriate to exclude missing outcome data that occurs due to patients who receive a transplant or die prior to the follow up period. Excluding these patients may lead to over/underestimating the intervention effect. It is important to find an appropriate method to account for these two events, such that an accurate estimation of the intervention effect can take place.

Objectives

This systematic review aims to evaluate current practice for dealing with missing outcome data due to patient mortality, kidney transplantation and other types of drop out. The review will appraise trials that use the KDQoL or SF-36 when measuring QoL. We focus on the KDQoL and SF-36 as these are commonly used in trials of dialysis patients[13]; however, the findings can be expanded to all QoL measures.

The proposed systematic review will answer the following questions.

1. Do trials including dialysis patients measuring QoL report how they deal with patients who die, have a transplant or are lost to follow-up?
2. What methods are currently used for dealing with patients who die, have a transplant or are lost to follow-up up in the analysis of trials?
3. How is the KDQoL reported and what statistical methods are used to analyse the QoL of patients?

METHODS

The systematic review will follow the Preferred Reporting Items for Systematic Reviews and Meta-Analyses (PRISMA) guidelines for reporting the results of systematic reviews [14]. The PRISMA-P protocol checklist for systematic review protocols was used when writing this protocol [15] and is provided in the supplementary information.

Peer-reviewed Literature

The search strategy will be developed by the primary author with the assistance of a specialist Health Sciences Librarian and peer-reviewed by an Honorary Consultant Nephrologist at the Leicester General Hospital. The electronic databases Medline, Web of Science, Cochrane Central Register of Controlled Trials (CENTRAL), Scopus and Cumulative Index of Nursing and Allied Health Literature (CINHAL) will be searched using combinations of keywords and topics. The main search strategy, written for Medline, is included in the supplementary information and will be adapted

For peer review only - http://bmjopen.bmj.com/site/about/guidelines.xhtml

for use in subsequent databases. Full search strategies will be included in the Appendix of the review. The databases will be searched from inception to 31/12/2020. Searches will be re-run before the final analysis, any studies published in the intervening period will be retrieved and included. The search will be limited to publications readily available in the English language. Search results will be exported and sorted using EndNote X9. All studies included will have their reference lists checked for additional studies.

Ongoing Studies

Trials registers are an important source for identifying additional randomised trials. ClinicalTrials.gov, the International Clinical Trials Register Search Portal (ICTRP), the European Union Clinical Trials Register (EUDRaCT) and the International Standard Randomised Controlled Trial Number (ISRCTN) will be searched to identify ongoing studies for inclusion in the review. Searches will be extensive to reduce the risk of publication bias.

Inclusion/Exclusion Criteria

For inclusion, the trial must be testing an intervention within a population of haemodialysis patients. The trial must measure QoL as an outcome. Trials must use the KDQoL (any variation) or the SF-36 (any variation) to measure QoL. Trials considered for inclusion will be phase 3 clinical trials of any design. Trials included will have recruited adult populations (18+) as the KDQoL and SF-36 are unlikely to be asked directly to children. There is no restriction on the setting or location of the trial. Articles must be either reported or readily available in the English language.

Screening

Titles, authors, abstracts, the date and location of publication will be exported into EndNote X9. Duplicates will be removed. Titles and abstracts will be screened for inclusion. The full-text articles will be screened for confirmation of suitability if the abstracts do not have sufficient information. Authors will be contacted if after full-text screening it is still not clear whether to include/exclude an article. All articles will be screened independently by two reviewers. Any disagreement on inclusion will be resolved by discussion or with another reviewer. A characteristics of excluded studies table will be presented in the Appendix of the review, detailing reasons for exclusion.

Data extraction

After screening, data extraction will be performed using a predetermined extraction form. Two data extraction forms will exist; one focusing on the approach used in completed trials; the other focusing on the planned approach for ongoing trials. Two reviewers will do the extraction, with differences discussed. A third reviewer will resolve if necessary. Pilot data extraction will test the forms prior to the main analysis.
Data to be extracted includes:

General

- Title

For peer review only - http://bmjopen.bmj.com/site/about/guidelines.xhtml

- Lead Author
- Year
- Author correspondence
- Study design
- Equivalent size control group
- Country
- Multi-centre (Y/N)
- Population
- Type of dialysis
- Research ethics obtained
- Informed consent obtained
- Inclusion of CONSORT PRIMSA flow diagram
- Number randomised
- Number analysed
- Number of dropouts, overall and by reason
    - Mortality
    - Transplant
    - Other reasons, for example due to an adverse event
- Time points of data collection

HRQoL specific

- HRQoL instrument used
- HRQoL primary outcome (Y/N)
- Methods used to account for missing HRQoL data
- KDQOL reporting methods
- KDQOL primary analysis method

Additional information for ongoing trials

- Status of trial
- Anticipated sample size
- Planned approach to main analysis (intention to treat, complete case, per protocol)
- Trial protocol available (Y/N)

Outcomes

- The number of studies that mention how death or transplants are dealt with in the analysis.
- Methods used to account for deaths and transplants in the analysis of quality of life.
- Most common methods used.
- Number of studies that fail to mention how they account for deaths and transplants in the analysis of QoL.
- Number of studies where death or transplants account for more than 5% of patients randomised.
- Associations between methods and the risk of bias.

Risk of Bias Assessment

For peer review only - http://bmjopen.bmj.com/site/about/guidelines.xhtml

RCTs are the gold standard of evidence-based medicine. However, they are open to bias if poorly designed or executed. When conducting a systematic review, in order to make reliable conclusions, the reviewers must consider the quality of the studies included. The Cochrane collaboration’s tool for assessing the risk of bias (RoB) [16] has been used in a significant number of previous systematic reviews. An updated version of this tool, the RoB 2.0 [17] will be used in this review to assess the RoB for each trial. Judgements about the RoB will be made independently by two reviewers and disagreements resolved through discussion.
There are five domains of bias, each with several elements. Each domain is concluded as “low”, “high” or “some concerning” level of risk. Completion of all domains leads to an overall risk of bias judgement. The aim of the quality assessment is not to further exclude studies from the review but instead to find the strengths and limitations of each study.

The sources of bias to be assessed are

- Domain 1: Risk of bias arising from the randomisation process.
- Domain 2: Risk of bias due to deviations from the intended interventions.
- Domain 3. Risk of bias due to missing outcome data.
- Domain 4: Risk of bias in measurement of the outcome.
- Domain 5: Risk of bias in the selection of the reported result.

For non-randomised controlled trials, the Cochrane RoB for non-randomised studies (ROBINS-I) [18] will be used to assess the quality of the trial. Similar to the RoB tool, the ROBINS-I will lead to an overall RoB judgement for each trial.

Patient and public involvement:

No patient involved. It was not appropriate or possible to involve patients or the public in the design, or conduct, or reporting, or dissemination plans of our research.

Data Synthesis

Completed trials

Completed trials that meet the inclusion criteria will be analysed using either RoB or ROBINS-I tool. The key information from the extracted data, alongside the RoB will be tabulated. Articles will be grouped into whether they have stated the methods used to deal with death and transplant patients. Articles that have will then be categorised into methods used. Authors of articles that have not will be contacted for this information. Responses will then be categorised into methods used or non-response. The results will be synthesised using descriptive statistics. Where possible, the percentage of patients who die or receive a transplant out of the total number of patients randomised will be calculated respectively for each trial. This review will highlight the common methods as well as the percentage of studies that fail to mention this issue entirely. Associations between reporting and the RoB will be assessed. The results of the review will be interpreted in line with the limitations (RoB) of each study.

Ongoing trials

For peer review only - http://bmjopen.bmj.com/site/about/guidelines.xhtml

The key information from the extracted data will be tabulated. Articles will be grouped into whether they have anticipated the methods they will use to deal with death and transplant patients. Articles that have will then be categorised into methods used. Authors of articles that have not will be contacted for this information.

Extensive systematic searches will strengthen the findings of this review. It is anticipated that the results of this review will identify the methods used to deal with patients who die or receive a transplant in the analysis of QoL. It is also anticipated that this review will identify that the methods used are inconsistent and the need to develop an acceptable method to be used consistently in nephrology trials.

Discussion

Previous papers highlight the general poor methodological quality of nephrology trials[19]. As well as this, papers have shown there is a mishandling of missing QoL data in a range of clinical areas[20]. A scoping review showed that there is potentially a high prevalence of trials of dialysis patients that do not include adequate methods, this review aims to show definitively how high the prevalence is.

The scoping review indicated some use of CCA in this discipline and the literature shows wide use of CCA in other clinical areas[21]. This review will assess whether this is the case for nephrology trials, where dealing with QoL data due to transplants is a unique issue. The limitations of CCA are that if patients have died or have dropped out due to adverse effects from the treatment, excluding them from the analysis could lead to an overestimation of the intervention and QoL estimates. Methods of imputation have also been implemented[20]; however, this is only justified if the missing data mechanisms are valid. If patients drop out due to the treatment, the assumption of the data being missing completely at random (MCAR) is invalid, and imputation methods cannot be employed. This review hopes to find other methods that have been used; if not, this review will highlight the need for more sophisticated methods to be introduced into the data analysis of clinical trials.

The information provided in this review will form the basis of further investigation into the most appropriate statistical methods of dealing with missing QoL data due to deaths, transplants and other reasons for drop out. Simply excluding these patients from the analysis in favour of complete case analysis (CCA) may lead to biased results, but anecdotal evidence suggests this maybe the most common approach used. Although the focus of this review is on kidney disease, the results will be applicable to any trials which include quality of life as an outcome.

Author Contributions: HW, LJG, NJC and JB were involved in all stages of writing the systematic review protocol and contributed to the concept and design, drafting, revision and approval of the final version. The search strategy for peer-reviewed literature and trial registers was created by HW and reviewed by JB and LJG.
Funding award: NIHR Applied Research Collaboration East Midlands
Funding: HW is funded by the National Institute for Health Research (NIHR) Applied Research Collaboration East Midlands (ARC EM). The views expressed are those of the author(s) and not necessarily those of the NIHR or the Department of Health and Social Care.
Data sharing statement: A summary of the extracted data will be available within the final publication.
Competing interests: None

For peer review only - http://bmjopen.bmj.com/site/about/guidelines.xhtml

ORCID IDs http://orcid.org/0000-0003-3958-0180

For peer review only

References

For peer review only - http://bmjopen.bmj.com/site/about/guidelines.xhtml

1. UK Renal Registry. UK Renal Registry 22nd Annual Report - data to 31/12/2018. Bristol, UK, 2020.
2. Find Specialist. Hemodialysis – NephCure Kidney International 2020 [Available from: https://nephcure.org/hemodialysis accessed 12/11/2020.
3. Kidney Care UK. Facts and Stats 2020 [Available from: https://www.kidneycareuk.org/news-and-campaigns/facts-and-stats/ accessed 26 October 2020.
4. Clarke AL, Yates T, Smith AC, et al. Patient's perceptions of chronic kidney disease and their association with psychosocial and clinical outcomes: a narrative review. *Clinical Kidney Journal* 2016;9((3)):494-502.
5. Novak JE, Inrig JK, Patel UD, et al. Negative trials in nephrology: what can we learn? *Kidney international* 2008;74(9):1121-27. doi: 10.1038/ki.2008.286
6. Natale P, Gutman T, Howell M, et al. Recruitment and retention in clinical trials in chronic kidney disease: report from national workshops with patients, caregivers and health professionals. *Nephrol Dial Transplant* 2020;35(5):755-64. doi: 10.1093/ndt/gfaa044
7. Baigent C, Herrington WG, Coresh J, et al. Challenges in conducting clinical trials in nephrology: conclusions from a Kidney Disease—Improving Global Outcomes (KDIGO) Controversies Conference. *Kidney international* 2017;92(2):297-305. doi: 10.1016/j.kint.2017.04.019
8. Tong A, Manns B, Hemmelgarn B, et al. Establishing core outcome domains in hemodialysis: report of the Standardized Outcomes in Nephrology–Hemodialysis (SONG-HD) consensus workshop. *American Journal of Kidney Diseases* 2017;69(1):97-107.
9. Hays RD, Kallich JD, Mapes DL, et al. Development of the Kidney Disease Quality of Life (KDQOLTM) Instrument. *Quality of life research* 1994;3(5):329-38. doi: 10.1007/bf00451725
10. Aiyegbusi OL, Kyte D, Cockwell P, et al. Measurement properties of patient-reported outcome measures (PROMs) used in adult patients with chronic kidney disease: A systematic review. *PloS one* 2017;12(6):e0179733-e33. doi: 10.1371/journal.pone.0179733
11. RAND. Kidney Disease Quality of Life Instrument 2020 [Available from: https://www.rand.org/health-care/surveys_tools/kdqol.html accessed 29 October 2020.
12. Macdougall IC, White C, Anker SD, et al. Intravenous iron in patients undergoing maintenance hemodialysis. *New England Journal of Medicine* 2019;380(5):447-58.
13. Cohen DE, Lee A, Sibbel S, et al. Use of the KDQOL-36™ for assessment of health-related quality of life among dialysis patients in the United States. *BMC nephrology* 2019;20(1):112-12. doi: 10.1186/s12882-019-1295-0
14. Moher D, Liberati A, Tetzlaff J, et al. Preferred reporting items for systematic reviews and meta-analyses: the PRISMA statement. *BMJ* 2009;339(jul21 1):b2535-b35. doi: 10.1136/bmj.b2535
15. Shamseer L, Moher D, Clarke M, et al. Preferred reporting items for systematic review and meta-analysis protocols (PRISMA-P) 2015: elaboration and explanation. *BMJ : British Medical Journal* 2015;349(jan02 1):g7647-g47. doi: 10.1136/bmj.g7647
16. Higgins JP, Altman DG, Gøtzsche PC, et al. The Cochrane Collaboration's tool for assessing risk of bias in randomised trials. *Bmj* 2011;343:d5928.
17. Sterne JAC, Savović J, Page MJ, et al. RoB 2: a revised tool for assessing risk of bias in randomised trials. *BMJ* 2019;366:l4898-l98. doi: 10.1136/bmj.l4898
18. Sterne JAC, Hernán MA, Reeves BC, et al. ROBINS-I: a tool for assessing risk of bias in non-randomised studies of interventions. *BMJ* 2016;355:i4919-i19. doi: 10.1136/bmj.i4919
19. Strippoli GFM. The Number, Quality, and Coverage of Randomized Controlled Trials in Nephrology. *Journal of the American Society of Nephrology* 2004;15(2):411-19. doi: 10.1097/01.asn.0000100125.21491.46
20. Fielding S, Ogbuagu A, Sivasubramaniam S, et al. Reporting and dealing with missing quality of life data in RCTs: has the picture changed in the last decade? *Quality of life research* 2016;25(12):2977-83. doi: 10.1007/s11136-016-1411-6
21. Fielding S, Maclennan G, Cook JA, et al. A review of RCTs in four medical journals to assess the use of imputation to overcome missing data in quality of life outcomes. *Trials* 2008;9(1):51-51. doi: 10.1186/1745-6215-9-51

For peer review only

For peer review only - http://bmjopen.bmj.com/site/about/guidelines.xhtml

For peer review only - http://bmjopen.bmj.com/site/about/guidelines.xhtml

For peer review only

Database: Medline 1946-date (includes epub ahead of print and in process citations. Updated daily)
Search Strategy:
----
1 Dialysis/ or dialysis.mp. (171204)
2 dialyses.mp. (821)
3 dialysate.mp. or Dialysis Solutions/ (13351)
4 dialysates.mp. (1416)
5 dialyzate.mp. (165)
6 dialyzates.mp. (28)
7 microdialysis.mp. or Microdialysis/ (17875)
8 microdialyses.mp. (4)
9 kidney replacement therapy.mp. (301)
10 kidney replacement therapies.mp. (27)
11 renal replacement therapy.mp. or Renal Replacement Therapy/ (15443)
12 renal replacement therapies.mp. (821)
13 artificial kidney*.mp. or Kidneys, Artificial/ (4751)
14 blood dialyser*.mp. (4)
15 blood dialyzer*.mp. (22)
16 Renal Dialysis/ (92294)
17 CAPD.mp. or Peritoneal Dialysis, Continuous Ambulatory/ (11179)
18 *dialysis/ (2279)
19 *dialyses/ (2279)
20 Hemofiltration/ or hemofiltration*.mp. (6341)
21 haemofiltration*.mp. (961)
22 ultrafilteration*.mp. (5)
23 biofilteration*.mp. (2)
24 1 or 2 or 3 or 4 or 5 or 6 or 7 or 8 or 9 or 10 or 11 or 12 or 13 or 14 or 15 or 16 or 17 or 18 or 19 or 20 or 21 or 22 or 23 (202167)
25 quality of life.mp. or "Quality of Life"/ (357611)
26 Qol.mp. (40312)
27 Hql.mp. (99)
28 Hqol.mp. (110)
29 H qol.mp. (8)
30 Hrqol.mp. (17377)
31 Hr qol.mp. (827)
32 life quality.mp. (7693)
33 value of life.mp. or "Value of Life"/ (6115)
34 quality adjusted life year*.mp. or Quality-Adjusted Life Years/ (18974)
35 QALY.mp. (9183)
36 Disability adjusted life year*.mp. (3584)
37 DALY.mp. (1935)
38 kidney disease quality of life-SF.mp. (5)
39 kidney disease quality of life.mp. (394)
40 kidney disease quality of life-36.mp. (47)
41 KDQOL*.mp. (350)
42 sf6.mp. (1658)
43 sf 6.mp. (476)
44 short form 6.mp. (111)
45 sf six.mp. (5)
46 short form six.mp. (56)
47 6-item short form.mp. (31)
48 six item short form.mp. (47)
49 sf12.mp. (410)
50 sf 12.mp. (4650)
51 short form 12.mp. (2187)

For peer review only

52 shortform 12.mp. (1)
53 sf twelve.mp. (1)
54 12-item short form.mp. (1245)
55 sf16.mp. (8)
56 sf 16.mp. (21)
57 short form 16.mp. (4)
58 sf sixteen.mp. (1)
59 short form sixteen.mp. (1)
60 16-item short form.mp. (13)
61 sf20.mp. (39)
62 sf 20.mp. (298)
63 short form 20.mp. (83)
64 sf twenty.mp. (7)
65 short form twenty.mp. (1)
66 20-item short form.mp. (31)
67 sf36.mp. (1313)
68 sf 36.mp. (21025)
69 short form 36.mp. (10446)
70 shortform 36.mp. (13)
71 sf thirty six.mp. (1)
72 short form thirty six.mp. (1)
73 36-item short form.mp. (4489)
74 SF-36.mp. (21025)
75 SF-6D.mp. (798)
76 SF-6.mp. (476)
77 SF-12.mp. (4650)
78 SF-20.mp. (298)
79 SF-16.mp. (21)
80 25 or 26 or 27 or 28 or 29 or 30 or 31 or 32 or 33 or 34 or 35 or 36 or 37 or 38 or 39 or 40 or 41 or 42 or 43 or 44 or 45 or 46 or 47 or 48 or 49 or 50 or 51 or 52 or 53 or 54 or 55 or 56 or 57 or 58 or 59 or 60 or 61 or 62 or 63 or 64 or 65 or 66 or 67 or 68 or 69 or 70 or 71 or 72 or 73 or 74 or 75 or 76 or 77 or 78 or 79 (382375)
81 24 and 80 (6724)
82 Randomized Controlled Trials as Topic/ (138501)
83 Clinical Trials as Topic/ (193802)
84 trial*.mp. (1756963)
85 clinical trial.mp. or Clinical Trial/ (721882)
86 randomi?ed control* trial.mp. (560831)
87 non randomised study.mp. (411)
88 randomised study.mp. (4303)
89 non randomized study.mp. (1307)
90 randomized study.mp. (26638)
91 82 or 83 or 84 or 85 or 86 or 87 or 88 or 89 or 90 (1762534)
92 81 and 91 (1251)

N=1,251

For peer review only

Reporting checklist for protocol of a systematic review.

Based on the PRISMA-P guidelines.

Instructions to authors

Complete this checklist by entering the page numbers from your manuscript where readers will find each of the items listed below.

Your article may not currently address all the items on the checklist. Please modify your text to include the missing information. If you are certain that an item does not apply, please write "n/a" and provide a short explanation.

Upload your completed checklist as an extra file when you submit to a journal.

In your methods section, say that you used the PRISMA-Preporting guidelines, and cite them as:

Moher D, Shamseer L, Clarke M, Ghersi D, Liberati A, Petticrew M, Shekelle P, Stewart LA. Preferred Reporting Items for Systematic Review and Meta-Analysis Protocols (PRISMA-P) 2015 statement. Syst Rev. 2015;4(1):1.

		Reporting Item	Page Number
**Title**			
Identification	#1a	Identify the report as a protocol of a systematic review	1
Update	#1b	If the protocol is for an update of a previous systematic review, identify as such	n/a
**Registration**			
	#2	If registered, provide the name of the registry (such as PROSPERO) and registration number	2
**Authors**			
Contact	#3a	Provide name, institutional affiliation, e-mail address of all protocol authors; provide physical mailing address of corresponding author	2

For peer review only

Contribution	#3b	Describe contributions of protocol authors and identify the guarantor of the review	7
**Amendments**			
	#4	If the protocol represents an amendment of a previously completed or published protocol, identify as such and list changes; otherwise, state plan for documenting important protocol amendments	n/a
**Support**			
Sources	#5a	Indicate sources of financial or other support for the review	7
Sponsor	#5b	Provide name for the review funder and / or sponsor	7
Role of sponsor or funder	#5c	Describe roles of funder(s), sponsor(s), and / or institution(s), if any, in developing the protocol	7
**Introduction**			
Rationale	#6	Describe the rationale for the review in the context of what is already known	3/4
Objectives	#7	Provide an explicit statement of the question(s) the review will address with reference to participants, interventions, comparators, and outcomes (PICO)	4
**Methods**			
Eligibility criteria	#8	Specify the study characteristics (such as PICO, study design, setting, time frame) and report characteristics (such as years considered, language, publication status) to be used as criteria for eligibility for the review	5
Information sources	#9	Describe all intended information sources (such as electronic databases, contact with study authors, trial registers or other grey literature sources) with planned dates of coverage	4/5
Search strategy	#10	Present draft of search strategy to be used for at least one electronic database, including planned limits, such that it could be repeated	9

For peer review only

Study records - data management	#11a	Describe the mechanism(s) that will be used to manage records and data throughout the review	5/6
Study records - selection process	#11b	State the process that will be used for selecting studies (such as two independent reviewers) through each phase of the review (that is, screening, eligibility and inclusion in meta-analysis)	5
Study records - data collection process	#11c	Describe planned method of extracting data from reports (such as piloting forms, done independently, in duplicate), any processes for obtaining and confirming data from investigators	5/6
Data items	#12	List and define all variables for which data will be sought (such as PICO items, funding sources), any pre-planned data assumptions and simplifications	5/6
Outcomes and prioritization	#13	List and define all outcomes for which data will be sought, including prioritization of main and additional outcomes, with rationale	6
Risk of bias in individual studies	#14	Describe anticipated methods for assessing risk of bias of individual studies, including whether this will be done at the outcome or study level, or both; state how this information will be used in data synthesis	5
Data synthesis	#15a	Describe criteria under which study data will be quantitatively synthesised	n/a
Data synthesis	#15b	If data are appropriate for quantitative synthesis, describe planned summary measures, methods of handling data and methods of combining data from studies, including any planned exploration of consistency (such as I2, Kendall's τ)	n/a
Data synthesis	#15c	Describe any proposed additional analyses (such as sensitivity or subgroup analyses, meta-regression)	n/a
Data synthesis	#15d	If quantitative synthesis is not appropriate, describe the type of summary planned	7
Meta-bias(es)	#16	Specify any planned assessment of meta-bias(es) (such as publication bias across studies, selective reporting within studies)	n/a

For peer review only

Confidence in cumulative evidence	#17	Describe how the strength of the body of evidence will be assessed (such as GRADE)	7

The PRISMA-P checklist is distributed under the terms of the Creative Commons Attribution License CC-BY 4.0. This checklist was completed on 04. December 2020 using https://www.goodreports.org/, a tool made by the EQUATOR Network in collaboration with Penelope.ai

For peer review only

PRISMA-P Checklist with information

SECTION AND TOPIC	ITEM NO	INFORMATION
**ADMINISTRATIVE INFORMATION**		
Title:		
Identification	1a	Measuring quality of life in trials including patients on dialysis: How are transplants and mortality incorporated into the analysis? A systematic review protocol
Update	1b	N/A
Registration	2	CRD42020223869
Authors:		
Contact	3a	➢ Hannah Worboys, hw315@leicester.ac.uk, Department of Health Sciences, University of Leicester, Leicester, UK.<br>➢ Professor Nicola Cooper, njc21@leicester.ac.uk, Department of Health Sciences, University of Leicester, Leicester, UK.<br>➢ Professor James Burton, jb343@leicester.ac.uk, Department of Cardiovascular Sciences, University of Leicester, UK.<br>➢ Professor Laura Gray, lg48@leicester.ac.uk, Department of Health Sciences, University of Leicester, Leicester, UK.
Contributions	3b	HW, LJG and NJC were involved in all stages of writing the systematic review protocol and contributed to the concept and design, drafting, revision and approval of the final version. The search strategy for peer-reviewed literature and trial registers was created by HW.
Amendments	4	N/A
Support:		
Sources	5a	This systematic review protocol is part of a PhD project being undertaken by HW and funded by the NIHR ARC EM.
Sponsor	5b	N/A
Role of sponsor or funder	5c	The funder had no role in the development of this protocol
**INTRODUCTION**		
Rationale	6	It is estimated that 25,000 people in the UK receive dialysis. Dialysis is an intrusive and time-consuming process that often causes significant reductions in quality of life. When enrolled in a clinical trial, it is estimated that 10% of patients will either die or receive a transplant within six months. It is unclear how these patients are dealt with in the analysis of quality of life measures. This review will assess current practice for dealing with transplants and mortality in trials of patients on dialysis.

For peer review only

Objectives	7	➢ Do trials of dialysis patients that measure the QoL report how they deal with patients who receive a transplant or die in the analysis?<br>➢ What methods are currently used for dealing with death/transplant in the analysis of dialysis trials?
**METHODS**		
Eligibility criteria	8	Phase 3 clinical trials of any design<br>Adult population, diagnosed with CKD, enrolled on dialysis<br>Incorporate measuring quality of life through use of the KDQoL or SF12
Information sources	9	**Peer-reviewed literature**<br>Medline, Web of Science , CENTRAL, CINAHL<br>Search databases from inception to 31/12/2020<br>Searched will be reran just before analysis<br>Limited to publications readily available in the English language<br>**Trial registers**<br>ClinicalTrials.gov, ICTRP, EUDRaCT, ISRCTN
Search strategy	10	Present draft of search strategy to be used for at least one electronic database, including planned limits, such that it could be repeated
Study records:		
Data management	11a	EndNote X9 will be used to store and manage and de-duplicate the references resulting from the searches and also used in the screening of titles and abstract process.
Selection process	11b	The reviewers (HW and GW) will independently screen the full texts of those studies and reports identified as having inclusion potential against the inclusion/exclusion criteria. Each stage of the screen process will be recorded using EndNote X9. Disagreement between the reviewers at all stages of screening is to be resolved by discussion and, if necessary, by the consultation of a third reviewer.
Data collection process	11c	Data extraction will be performed independently by two reviewers (HW and GW) using a pre-tested extraction form. Any missing information will be sought by contacting the authors. Disagreement between the reviewers at all stages of screening and data extraction is to be resolved by discussion and, if necessary, by the consultation of a third reviewer
Data items	12	General<br>➢ Author<br>➢ Trial name<br>➢ Publication date and location<br>➢ Intervention and control<br>➢ Trial design

For peer review only

		➢ Trial date and location<br>➢ Approach to the main analysis<br>➢ Sample size<br>➢ Primary outcome<br>➢ QoL measure used (KDQoL or RAND short form)<br>➢ Follow-up period<br>Specific information relating to death/transplants<br>➢ Death mentioned (Y/N)<br>➢ Transplant mentioned (Y/N)<br>➢ Number of deaths stated<br>➢ Number of transplants stated<br>➢ The method used to account for deaths in the analysis of QoL<br>➢ The method used to account for transplant in the analysis of QoL
Outcomes and prioritization	13	➢ Different methods used to account for deaths and transplants in the analysis of quality of life.<br>➢ Most common methods used<br>➢ Number of studies that fail to mention how they account for deaths and transplants in the analysis of quality of life
Risk of bias in individual studies	14	RoB 2.0 for randomised trials<br>ROBINS-I for non-randomised trials
Data synthesis	15a	n/a
	15b	n/a
	15c	n/a
	15d	Narrative summary of trials
Meta-bias(es)	16	N/A
Confidence in cumulative evidence	17	The results will be interpreted in line with the limitations (RoB) of the study

*** It is strongly recommended that this checklist be read in conjunction with the PRISMA-P Explanation and Elaboration (cite when available) for important clarification on the items. Amendments to a review protocol should be tracked and dated. The copyright for PRISMA-P (including checklist) is held by the PRISMA-P Group and is distributed under a Creative Commons Attribution Licence 4.0.**

From: Shamseer L, Moher D, Clarke M, Ghersi D, Liberati A, Petticrew M, Shekelle P, Stewart L, PRISMA-P Group. Preferred reporting items for systematic review and meta-analysis protocols (PRISMA-P) 2015: elaboration and explanation. BMJ. 2015 Jan 2;349(jan02 1):g7647.

For peer review only
